# Prevalence of U.S. Pregnant Women Meeting 2015 ACOG Physical Activity Guidelines

**DOI:** 10.1016/j.amepre.2016.05.023

**Published:** 2016-09

**Authors:** Kathryn R. Hesketh, Kelly R. Evenson

**Affiliations:** 1 Department of Epidemiology, Gillings School of Global Public Health, University of North Carolina–Chapel Hill, Chapel Hill, North Carolina; 2 Population, Policy and Practice, UCL Institute of Child Health, London, United Kingdom; 3 Center for Health Promotion and Disease Prevention, University of North Carolina–Chapel Hill, Chapel Hill, North Carolina

## Introduction

In December 2015, the American College of Obstetrics and Gynecology (ACOG) published updated physical activity (PA) guidelines for pregnant women, recommending women with uncomplicated pregnancies engage in ≥20–30 minutes/day of exercise on most days/week.^1^ Previous guidelines advocated ≥30 minutes/day of exercise on most days/week^2^; updated recommendations represent a more obtainable PA target during pregnancy. Although previous estimates indicated that few women in the U.S. accrued sufficient PA during pregnancy,^3^ it is important to determine how many women meet new recommendations. This paper describes the prevalence of pregnant women meeting the 2015 ACOG PA guidelines.

## Study Design

Data were from a population-based survey assessing the U.S. population’s health, the National Health and Nutrition Examination Survey (NHANES) (collected 2007–2014^4^; analyzed February 2016). PA was self-reported during interviews with 247 pregnant women aged 20–44 years, using a questionnaire.^5^, ^6^ Minutes/week women spent in moderate and vigorous leisure-time PA (LTPA) and in active transport (walking/cycling) during a typical week were derived. The number of days women engaged in weekly PA overall and by trimester of pregnancy (reported in 2007–2012 only; *n*=131) was calculated. Guidelines were operationalized as ≥100 or ≥150 minutes/week LTPA (i.e., ≥20 or ≥30 minutes on ≥5 days/week), with and without the requirement of “on most (5) days/week.” Whether including weekly active transport influenced the prevalence of women meeting PA guidelines was also explored. All estimates were weighted to reflect the U.S. population. NHANES participants gave informed written consent; the University of North Carolina IRB approved this study.

## Results

Pregnant women were aged 28.8 (SD=6.0) years on average and had a mean BMI of 29.6 (SD=8.0); 65% women had some/college education, and 51% were of non-white ethnicity. Sixty percent of women reported engaging in no LTPA; those who did (*n*=148) were active for a median 150 (interquartile range [IQR], 80–240) minutes/week, which decreased by trimester (T1_*n*=19_: 180 [60–300]; T2_*n*=15_: 120 [60–150]; T3_*n*=18_: 97.5 [60–200]; Kruskal–Wallis test, *p*=0.34). Those engaging in active transport (*n*=81) did so for a median 140 (60–270) minutes/week (T1_*n*=17_: 180 [60–360]; T2_*n*=15_: 180 [60–360]; T3_*n*=14_: [60–270]; *p*=0.63]. The proportion of women meeting ACOG PA guidelines ranged from 12.7% to 45.0%, depending on operationalization (Table 1), and did not differ by trimester among the subsample. There was little difference in prevalence of women meeting guidelines when ≥5 days was specified (12.7%–13.3%). Ignoring “most days,” including women with any ≥100 minutes/week or ≥150 minutes/week of LTPA resulted in prevalences of 28.9% and 23.4%, respectively. Inclusion of active transport was associated with higher prevalence of meeting guidelines.

## Conclusions

The updated ACOG guidelines recognize the benefits of PA during pregnancy,^1^ providing women with a more manageable target of ≥20 minutes of exercise on most days/week. Yet, most U.S. pregnant women reported LTPA below this target, in part explaining why the few women meet guidelines, regardless of trimester. Interestingly, using the thresholds of ≥100 versus ≥150 minutes/week made little difference to the proportion of women meeting guidelines, but removing the need to engage in PA on ≥5 days/week resulted in approximately double the number of women meeting guidelines. In current ACOG guidelines, active transport does not contribute to “exercise,” yet makes a meaningful contribution to women’s overall PA, mainly through walking.^7^ Consequently, including active transport in women’s weekly PA had a substantial impact on the proportion of women classified as meeting recommendations.

Although new ACOG guidelines were largely based on research using self-report measures, self-reported PA is often higher than accelerometry.^8^ Findings here may therefore represent an overestimation of women meeting PA guidelines. As NHANES ceased to oversample pregnant women during the study period, this study combined data from 2007 to 2014, preventing analysis of trends. It was also unable to identify women for whom PA was contraindicated.

Nevertheless, practitioners face a considerable challenge in ensuring that pregnant women free of complications meet PA guidelines. However, practitioners are well placed to highlight the benefits of PA; to assess PA readiness (using resources like the PA Readiness Medical Examination,^9^ a guideline for health screening prior to exercise participation); and advise on how to exercise safely during pregnancy. In light of these findings, emphasizing the need to be physically active across the week and the various types of activity (e.g. active transport) that contribute toward daily PA (where not contraindicated) may also be helpful,^10^ as PA does not have to be limited to planned exercise. Finally, given the current extent of low PA during pregnancy, multilevel approaches (e.g., intervening at the individual, interpersonal, and community levels simultaneously) will likely best support pregnant women and encourage them to achieve PA recommendations.

## Figures and Tables

**Table 1:** Prevalence of Pregnant Women Meeting Updated ACOG 2015 Physical Activity Guidelines (*n*=247); NHANES 2007–2014

**Physical activity threshold**	**% (*****n*****)**	**95% CI**
Lower physical activity threshold^a^^,^^b^		
≥100 minutes of LTPA on ≥5 days/week	13.1 (29)	8.0, 20.6
Any ≥100 minutes of LTPA during week	28.9 (67)	24.2, 34.1
≥100 minutes of LTPA or active transport on ≥5 days/week	13.3 (30)	8.7, 19.8
Any ≥100 minutes of LTPA or active transport during week	45.0 (110)	37.5, 52.6
Higher physical activity threshold^b^^,^^c^		
≥150 minutes of LTPA on ≥5 days/week	12.7 (27)	6.9, 22.2
Any ≥150 minutes of LTPA during week	23.4 (52)	14.9, 34.9
≥150 minutes of LTPA or active transport on ≥5 days/week	12.9 (28)	7.5, 21.3
Any ≥150 minutes of LTPA or active transport during week	37.8 (89)	32.6, 43.3

*Note:* LTPA defined as a moderate or vigorous intensity for ≥10 minutes; Active transport defined as walking or cycling for ≥10 minutes.

ACOG, American College of Obstetrics and Gynecology; LTPA, leisure-time physical activity; NHANES, National Health and Nutrition Examination Survey.

a Calculated by multiplying the lower 20-minute threshold by 5, where 5 is deemed to be “most” days.

b No significant difference in women meeting PA guidelines by trimester (T1: *n*=39; T2: *n*=46; T3: *n*=46, χ^2^ test) for lower and higher physical activity thresholds, regardless of inclusion of “any” physical activity or active transport.

c Calculated by multiplying the higher 30-minute threshold by 5, where 5 is deemed to be “most” days.
